# Emotions and reading: When reading is the best way to improve skills in adolescents

**DOI:** 10.3389/fpsyg.2023.1085945

**Published:** 2023-02-06

**Authors:** Elena del Pilar Jiménez-Pérez, María Isabel de Vicente-Yagüe Jara, Manuel León Urrutia, Pedro García Guirao

**Affiliations:** ^1^Department of Didactics of Language, Art and Sports, University of Málaga, Málaga, Spain; ^2^Department of Didactics of Language, University of Murcia, Murcia, Spain; ^3^Department of Computer Science, University of Southampton, Southampton, United Kingdom; ^4^Department of Linguistics, WSB University in Wroclaw, Wroclaw, Poland

**Keywords:** emotional intelligence, reading competence, adolescents, quantitative research, reading habits, reading intervention

## Abstract

In recent years much research on reading competence in different languages has been published in parallel with the interest generated by the results of the PISA and PIRLS reports which were disseminated in the media and which have subsequently garnered the attention of public authorities. Studies that relate reading competence with emotional intelligence, however, are less frequent. This study aims to deepen the relationship between both constructs, using a quasi-experimental longitudinal approach that observes the evolution of 389 high school students in Spain from 16 to 18 years old. Evidence of a direct relationship between reading competence and emotional intelligence was obtained, particularly in the experimental group in which reading habits were stimulated.

## Introduction

Reading competence (RC) and emotional intelligence (EI) constructs have been widely studied in the scientific literature in recent years, as well as their importance in the field of education. Reading competence is a skill that can be specifically taught for better development, but it is a competence which is refined naturally through a person’s reading habits since readers with stronger reading habits score better in reading skills (Jiménez-Pérez, 2014; OECD, 2016): training as a receiver qualifies as an issuer (Echeverría, 1998). In addition, it has been demonstrated that reading for pleasure provides students with a better ability to get good grades (Sullivan and Brown, 2015), that reading lengthens life (Bavishi et al., 2016), and that reading books outside of class when at the school stage of life predicts professional success in a positive way (Taylor, 2016). Reading also offers a long list of advantages such as reducing stress, improving behavior, and helping people to empathize and to make more creative decisions (Rodríguez, 2016).

With regard to reading competence and comprehension, there is still a long way to go in terms of research. For example, it should be noted that there is no scientific literature on the improvement of reading comprehension in Spanish since “not even serious attempts have been made to make this identification through reviews of the previous research” (Ripoll Salceda, 2014). Furthermore, there are other issues related to reading habits such as, for example, the impact of information and communication technologies (ICT) on people’s ways of reading involves “the need to attend to new visual skills and to new reading skills” (Sánchez-Claros, 2016), or that motivating reading by awakening curiosity is more effective than using reading or motivation strategies (Gurning, 2017), such as through ICT, which facilitate the general acquisition of professional and personal competences (Martínez-Cerdá and Torrent-Sellens, 2017) and thereby stimulate higher involvement on the part of students than they had before the digital world (Alonso-Mosquera et al., 2016). Therefore, if reading habits change, either in the devices for reading (Cordón-García, 2018), the typology of digital texts, or the brain’s changing way of learning under technological impacts, reading competence must also be affected to a greater or lesser extent (Amiama-Spaillat and Mayor-Ruiz, 2017).

On the other hand, it has been demonstrated that emotions emerge during the reading of, for example, fictional literature. Thus, we agree with Vega et al. (1996, p. 304) that “readers are able to represent accurately the implicit emotional states of protagonists as a consequence of understanding their actions, goals, and relationships with other characters” (Gernsbacher et al., 1992; Gernsbacher and Robertson, 1992; Vega et al., 1996). Other works propose that texts of children’s and youth literature be directed to the understanding, regulation, and management of emotions (Vicente-Yagüe Jara, 2018). Sometimes, reading also makes it possible to work on the emotions stemming from grief with students in early and primary education courses (Vicente-Yagüe Jara, 2018). Although when dealing with learning-related reading, emotions also appear, for example, “when the text becomes too difficult or easy for the reader and when conceptual obstacles create cognitive disequilibrium” (Graesser and D'Mello, 2012). It has even been shown that poor literacy is closely associated with states of anxiety (Stevenson et al., 2018).

There is also an inverse path whereby emotions influence reading: “when emotions are evoked during reading, they are likely to have an immediate influence on the reading process” (Mar et al., 2011). This is perhaps in part because “language as an expression of different personalities” (Vilches, 2004) converges in written texts through reading comprehension, and both self-concept and self-esteem also influence the degrees of reading comprehension (Asadi, 2014). In addition, there is evidence of a positive relationship between mood and comprehension performance (Mengual, 2017). To frame the concept, we agree that emotional intelligence or affective competence is defined as “the ability to recognize, express and channel emotional life. Personal balance, self-esteem and empathy acquire special importance. The meta-affectivity or ability of the subject to know and govern feelings is also important” (Martínez-Otero-Pérez and García-Lago, 2013).

Therefore, the proper management of emotions influences academic performance as well as influencing group performance (Suverbiola-Ovejas, 2011) with the result that this is better as a higher emotional intelligence score is obtained (Extremera and Fernández, 2004; Páez and Castaño, 2015; Pishghadam et al., 2022) according to the IE-stress relationship pointed out by Bermejo-Casado et al. (2017), who highlight the key role of reading competence as one of the main academic skills (Bermejo-Casado et al., 2017; Pishghadam et al., 2022). It is a skill which even influences cognitive aspects such as memory (Barreyro et al., 2009; Schamalbach, 2016; Vernucci, 2017) since, not in vain, the term “intelligence” comes from Latin *intellegere*, and it is composed of *inter* + *legere* (reading) (Nouwens et al., 2018). In this way, the relationship between EI and reading is verified in numerous investigations (Freitas, 2012; Valdez, 2017), thereby demonstrating the direct relationship between a greater love of reading and a greater reading habit (De Nóbrega and Franco, 2016) and higher EI scores. Nevertheless, despite these investigations that defend the relationship between both constructs (Jiménez-Pérez et al., 2019; Akbari and Pishghadam, 2022), others have concluded that “although reading-comprehension proficiency was relatively associated with several dimensions of EQ (intrapersonal, interpersonal, and stress management), the total EQ and its subscales were found to be poor predictors of reading comprehension” (Jiménez-Pérez et al., 2019). Further research is therefore necessary.

A better knowledge of the relationship between both constructs can facilitate changes in countries’ educational regulations aimed at having a significant decrease in school failure rates, as it is already known that the development of personalized strategies according to student preferences improves reading comprehension (Akbari and Pishghadam, 2022). To contribute to this knowledge, and within this scheme of intervention strategies that can be put into practice to improve academic performance in the classroom, (Fernández Millán et al., 2021; Jiménez-Pérez, 2022) this study aims to analyze the effect of a motivational strategy for reading with a classroom program of intervention conducted by teachers and based on the constructs of reading competence and emotional intelligence. For this, two groups of students were selected to be followed from the beginning to the end of their high school studies (two academic years). In one group, students received specific instruction on reading from their teachers, and the other group followed the regular curricular program established in the official regulations.

The hypothesis to be tested is as follows: students who receive specific training in reading motivation will demonstrate better performance in both reading competence and have an improvement in their emotional intelligence scores.

## Method

### Participants

The study involved 389 students aged between 14 and 16 years of age, of which 45% were boys and 55% girls, belonging to 12 classes from six public high schools located in middle-class areas in Andalusia (Spain). The students were divided into centers at which they either did not receive any type of intervention (the control group or alpha group), and those at which the promotion of reading was carried out (omega group). In the omega group, teachers controlled the number of readings and type of reading after a brief approach through various media (viewing of films, getting to know the authors, and anecdotes about the literary movement of each work).

The alpha group consisted of 193 (86 male and 107 female, 44.6 and 55.4% respectively) and the omega group consisted of 196 (89 male and 107 female, 45.4 and 54.6% respectively).

### Instruments

Two evaluation instruments were selected, both scientifically guaranteed for their reliability and validity from a psychometric point of view: the Wong and law emotional intelligence scale (WLEIS) scale (Pishghadam and Shayesteh, 2017) to measure emotional intelligence, and the CompLEC reading competence test (Ghabanchi and Rastegar, 2014).

### Wong and law emotional intelligence scale

This scale provides a measure of basic self-reporting of emotional intelligence, and was initially developed for the organizational field. The recommended age to perform this test is from 16 years onwards, so it is valid for the target group chosen for this study. The version of the WLEIS in Spanish of Vila and Pérez-González (2007) validated by Merino et al. (2016) has been used (Wong and Law, 2002; Balci, 2017), although recently a new Spanish version WLEIS-S (Wong and Law, 2002) has become made available. It is composed of 16 items measured using a 7-point Likert-type scale from strongly agree to strongly disagree where, in general, a higher score corresponds to greater emotional intelligence. These items are distributed according to four dimensions (with four items per dimension): (1) self-emotion appraisal; (2) others’ emotion appraisal; (3) uses of emotion, and (4) regulation of emotion. This allows for adequate internal consistency values for each subscale to be obtained.

### The CompLEC reading competence test

This test consists of five texts, three continuous and two discontinuous ones, and a total of 20 questions prepared following the PISA 2000 (OECD, 2016) parameters Llorens et al. (2011). The extent of the continuous texts varies from 274 to 426 words, and the written texts are mainly expository and argumentative. The discontinuous texts contain paragraphs, graphs and diagrams, with a smaller scope of 130 words.

The 20 questions are divided, following PISA guidelines, into three categories that represent the three basic aspects of reading competence that PISA evaluates: (a) 5 information retrieval questions; (b) 10 integration questions; (c) and 5 questions that reflect on the content and form of the text. The response format for 17 items is multiple choice with four alternatives, and three are open questions that require a brief response from the student. The psychometric properties of this test have been adequately contrasted in terms of reliability, homogeneity, difficulty and validity.

Although this test has been designed for adolescents between 11 and 14 years of age, the texts and questions used are perfectly valid for higher age ranges since the type of texts and questions suggested by PISA have been used. The CompLEC test can be obtained at the Ministry of Education of the Government of Spain’s webpage, www.lee.es.

### Research design and procedure

In this research, a post-descriptive quasi-experimental and longitudinal design was used. The control group followed, without alteration, the current official curriculum of the Junta de Andalucía government established for high school studies. Four start and end measurements (pre-test—post-test) framed in the two intervention periods (one in each course) were carried out in the group (omega) in which the experiment was conducted. Thus, each student completed both the IE test and the reading test at the beginning and at the end of the first year and the second year of high school, with the same tests being used in all cases and for both groups, alpha and omega. *The study, which involved minor human participants, was reviewed and approved by the Institutional Review Board of Asociación Española de Comprensión Lectora the Spanish association of reading comprehension. Written informed consent to participate in this study was provided by the parents or legal guardian of the minor participants.*

To ensure homogeneity in the collection of data, in both groups the individual follow-up for each student was conducted anonymously during class time, in the usual classroom with the collaboration of each center’s language and literature teachers, who had received the same instructions with respect to the intervention group. Likewise, the ethical premises required for research with human beings were respected with regard to informed consent, the guaranteed right to information and protection of personal data, confidentiality and freedom from discrimination for any reason, and the freedom to withdraw at any time.

A descriptive analysis (means and standard deviations) was proposed in each construct (IE and RC), for both groups and sexes, as well as a mixed ANOVA for repeated measures, in which the initial-final evaluation factors were included (P1–P4) according to group and sex typology.

### Intervention

In the alpha group, the official regular teaching-learning process was not altered. The intervention project was launched in the omega group. For the students in the omega group, 30 books were chosen at random as a reading proposal from lists of the best books in the world in Spanish from a Google random query. The lists were from Wikipedia, Quelibroleo, culturacolectiva, 20 min, etc. and excluded those that could present explicit adult content (violence and sex).

The selected books (all in Spanish) were: Exupéry’s El Principito (The Little Prince), Boyne’s El Niño del Pijama de Rayas (The Boy in the Striped Pajamas), Ende’s La Historia Interminable [The Endless Story, Bécquer’s Rimas (Rhymes), Wilde’s El Retrato de Dorian Gray (The Picture of Dorian Gray), Poe’s El Escarabajo de Oro (The Gold-Bug), Galdós’ Tormento (Torment), Asimov’s Amigos Robots (Robbie), Mihura’s Tres Sombreros de Copa (Three Top-Hats), Reverte’s La Carta Esférica (The Spherical Letter), Ovid’s La Metamorfosis (The Metamorphoses), Lorca’s La Zapatera Prodigiosa (The Prodigious Shoemaker), Jiménez’s Platero y Yo (Platero and I), Orwell’s Rebelión en la Granja (Animal Farm: A Fairy Story), Delibes’ El Príncipe Destronado (The Deposed Prince), Bazán’s Insolación (Insolation), Borges’ El Aleph (The Aleph and Other Stories), Bécquer’s Leyendas (Legends), Shakespeare’s Mucho Ruido y Pocas Nueces (Much Ado About Nothing), Ábalos’ Grimpow, Hurley’s Ghostgirl, Rowling’s Harry Potter 7 (Harry Potter and the Deathly Hallows), Tolkien’s El Hobbit (The Hobbit), Zafón’s Marina, Mendoza’s El Misterio de la Cripta Embrjada (The Mystery of the Haunted Crypt), Galeano’s Mitos (Myths), Fences’ Soldados de Salamina (Soldiers of Salamina), Sierra i Fabra’s Las Chicas de Alambre (The Wire Girls), Moccia’s A Tres Metros Sobre el Cielo (Three Meters Above the Sky), and Espinosa’s El Mundo Amarillo (The Yellow World)].

The intervention was carried out in 30 sessions of 60 min each, giving a total of 30 readings (five per quarter, fifteen per course) since it has been proven that planning the actions improves reading comprehension (Vila and Pérez-González, 2007). The motivation to read was implemented through tabs with data from the films (when there was a film version of the book), curious facts about the authors and anecdotes about the literary movements to which they belong (for example, that Galdós and Bazán maintained an idyll out of wedlock, that Ende abhorred the film that was made of his book and denounced the production company, or that the causes of Poe’s premature death are yet to be resolved).

As an example, we summarize an intervention with “Leyendas,” a book by the Spanish writer Gustavo Adolfo Bécquer. At the beginning of the session, for 5 min, several anecdotes are told, the most interesting being that the author himself predicted his posthumous fame with the phrase “I have a feeling that I will be more and better known dead than alive” since it is the best way to introduce a romantic reading about superstitions, souls and deaths.

Also that he was orphaned and found his love for reading in his godmother’s library, that he suffered from sexually transmitted diseases, that he died very young or that his writings were mysteriously lost. The teacher begins reading aloud for another 5 min, to focus attention on the reading. The remaining 50 min pass in silence because we have already verified that if they read aloud they are more concerned with appearing to read better than they actually do. At the end of the session, the teacher asks several random students to find out how far they have progressed (13 pages on average, 3.5 min/page). The readings are continued at home at a rate of 18 days maximum to conclude it. The trailer Bécquer and the witches of Decine21 is uploaded to the teaching platform.^1^

## Results

Tables 1, 2 show the mean and standard deviation of the variables emotional intelligence (EI) and reading competence (RC) in the four evaluation phases corresponding to the control group and the intervention group, as well as the differences between the sexes of the students participating in each phase.

**Table 1:** Descriptive statistics.

		Control group (alpha)		Intervention group (omega)	
		*M*	*SD*	*M*	*SD*
EI	P1	4.12	0.22	4.05	0.21
	P2	4.31	0.18	4.36	0.40
	P3	4.32	0.25	4.79	0.30
	P4	4.54	0.24	5.13	0.16
RC	P1	12.1	0.28	11.1	0.29
	P2	13.7	0.65	12.3	0.62
	P3	13.5	0.18	13.8	0.51
	P4	13.6	0.22	15.6	0.45

**Table 2:** Mean per group and sex.

		Control group (alpha)		Intervention group (omega)	
		Female	Male	Female	Male
EI	P1	4.18	4.05	4.12	4.19
	P2	4.32	4.30	4.33	4.39
	P3	4.39	4.25	4.81	4.75
	P4	4.58	4.49	5.14	5.12
RC	P1	12.2	12.0	11.5	10.6
	P2	13.7	11.7	12.7	11.7
	P3	12.3	12.7	13.9	12.7
	P4	13.6	13.6	15.7	15.5

### Reading competence

The results for the participants in the intervention program show improvement over the course of the 2 years and along the four evaluations. The mixed ANOVA indicates a significant effect [*F*(_1,41_) = 29.11, *p* < 0.001], as well as a positive intragroup impact for the evaluation factor [*F*(_1,41_) = 35.58, *p* < 0.001].

Regarding the *post hoc* tests, in the omega group (intervention) there were significant differences between the ranges of P1 to P2 (*p* < 0.001) and of P3 to P4 (*p* < 0.001). However, in the alpha group (control) the differences were significant only in the range P1 to P2 (*p* < 0.001), while they were not significant in the range P3 to P4 (*p* > 0.001). In contrast, the total scores (P1 to P4) differed positively in both groups (alpha and omega), and were significant in the two groups (*p* < 0.001). Similarly, significant differences were found between the intervention (omega) and control (alpha) groups where the main effect of the group factor was found [*F*(_1,63_) = 15.69, *p* < 0.01]; between both groups there is a difference of two points in favor of the omega intervention group. The effects of intervention and group in favor of the omega group were also significant, which together with the *ad hoc* tests confirm these significant differences. However, the size of the effect is moderate, so the magnitude of the differences is not highlighted by its amplitude (*d* = 0.58). Finally, the differences were not significant in the sex factor, neither with respect to the group (*p* = 0.285) nor the comparative evaluation/group/sex.

In general, reading competence improved in both groups (alpha: 12.1–13.6, omega: 11.1–15.6), with the biggest difference being in the omega group with a total of four points out of 20 against a point and a half from alpha, and starting from an initial level of one point less in the omega group.

### Emotional intelligence

Participants in the intervention group (omega) also improved their emotional intelligence results over the 2 years and the four evaluations. The ANOVA analysis shows evidence of major effects in the intervention [*F*(_1,41_) = 61.42, *p* < 0.001]. A positive effect can also be observed in intragroup analysis with evidence of significant differences in the omega group from the P1 to P2 ranges (*p* < 0.001) and from P3 to P4 (*p* < 0.001). However, in the control group (alpha), the differences were not significant between the ranges P1–P2 and P3–P4, although they were in the totals P1–P4. In both cases there was a significant overall improvement (*p* < 0.001) which, in the case of the omega group, reached an improvement of 1.08 total of a maximum score of seven.

Finally, as with reading competence, emotional intelligence did not show significant differences in the sex factor or in combination with the other comparatives (group and intervention).

## Discussion

In this research the objective was to analyze how an intervention project to promote reading can influence an improvement in students’ reading competence and emotional intelligence since a review of the literature points to a direct relationship between both constructs.

The results obtained show that the intervention group (omega) scored significantly above the control group both in reading competence as well as in emotional intelligence, offering evidence of compliance with the hypothesis proposed in this research. These data highlight the fact that specific instruction in the field of reading through the three chosen aspects (parallelism with the cinema, anecdotes about the author, and curiosities about the literary movement to which the work belongs) results in an improvement in the score of both the emotional intelligence and reading competence of the students in an intervention program developed over 2 years. The adoption of this hypothesis has special repercussions in combination with the theses on group work and teaching exposed by Vidal Raméntol and Fuertes Camacho (2013) relative to the teacher taking the emotions of a student into consideration and to the horizontal communication in the classroom (Merino et al., 2016).

In the evaluated phases it was observed that, although both groups increased their scores in general, the intervention group (omega) improved more than the control group (alpha) in both reading competence and emotional intelligence. This indicates that although the curriculum provided by the Junta de Andalucía fulfills its functions, the results can be significantly improved by planning a basic program to promote reading as proposed in this study. With regard to sex, the differences were positively inclined toward the female sex, which scored significantly better in both reading and emotional intelligence in the last phase of evaluation, as evidenced by the improvement not only in the intervention group (omega) but also in the control (alpha).

It is necessary to highlight that among the limitations of research designs with repeated measures there exists the possible effect of learning by practice since students have been able to improve their responses with repetition, and data obtained in later stages may seem better without really being so. In this case, however, it can be considered that the potential effect of this bias was limited both by the age of the participants and by the time difference over which the measurement instruments were administered. In addition, after each administration there was no feedback to students, so it is unlikely that they could “learn” which options or measures were more correct than others and try to improve their scores. Therefore, it is reasonable to consider that the improvements obtained in both reading competence and emotional intelligence are due more to the reading stimulus intervention program than to a bias derived from the experience effect with the measuring instruments.

This research provides a basis from which to propose a generalized program of intervention in reading promotion in Spanish educational centers in order to improve two of the most studied skills in recent years in the field of education: reading competence and emotional intelligence. On the one hand, a program could seek to stimulate students’ own resources to optimize their level of reading competence (Vidal Raméntol and Fuertes Camacho, 2013; Extremera et al., 2019) within the existing seven levels: literal (objective and subjective), representative, inferential, critical, emotional, creative and meta-cognitive (Jiménez-Pérez, 2015). And on the other hand, it could seek to perfect personal skills in emotional intelligence (Alfonso et al., 2017): the conception of one’s own emotions, the ability to control them, the ability to self-motivate (tremendously useful in the field of teaching), the recognition of others’ emotions and the control of relationships.

It would be interesting to extend this research to students from other Spanish-speaking countries, such as those in Latin America, in order to evaluate reading competence comparatively across the broad spectrum that the Spanish language and its cultural nuances present in the world. In addition, it would be interesting to carry out a comparative analysis of the curricular programs in the different countries where Spanish is the official language according to the parameters analyzed in this study. It is also an interesting line of research to check the opposite effect, that is, to test whether implementing a program for improvement in emotional intelligence in both primary and secondary school classrooms can improve academic performance in reading competence so that in the long run it would not be necessary to train such competence at higher educational levels, such as at university.

## Data availability statement

The raw data supporting the conclusions of this article will be made available by the authors, without undue reservation.

## Ethics statement

The study, which involved minor human participants, was reviewed and approved by the Institutional Review Board of Asociación Española de Comprensión Lectora, the Spanish association of reading comprehension. Written informed consent to participate in this study was provided by the parents or legal guardian of the minor participants.

## Author contributions

EJ-P came up with the idea and focused on the theoretical framework. ML focused on the methodology. MV-YJ focused on the discussion and conclusions. PG focused on the writing and the translation into English. All authors contributed to the article and approved the submitted version.

## Conflict of interest

The authors declare that the research was conducted in the absence of any commercial or financial relationships that could be construed as a potential conflict of interest.

## Publisher’s note

All claims expressed in this article are solely those of the authors and do not necessarily represent those of their affiliated organizations, or those of the publisher, the editors and the reviewers. Any product that may be evaluated in this article, or claim that may be made by its manufacturer, is not guaranteed or endorsed by the publisher.

## Footnotes

^1^ https://www.youtube.com/watch?v=0Kxajyp4v60
